# Does ‘giving’ influence governance? Application of a framework describing the role of private philanthropy in Indian national health priority setting

**DOI:** 10.1186/1753-6561-6-S5-P5

**Published:** 2012-09-28

**Authors:** Devaki Nambiar, Prasanna Saligram, Kabir Sheikh

**Affiliations:** 1 Public Health Foundation of India, New Delhi, India

## Introduction

Even as philanthropy has played a role in financing public health for centuries, the past few years have seen a significantly increased investment in the health sector in low- and middle-income (LMIC) settings through the interventions of large private philanthropic organizations. This change has been accompanied by widespread tacit approbation in the community of beneficiary academicians and institutions and, some limited examination and criticism of the role that these stakeholders have played in influencing health priorities within nations. The critical voice is important, but has been limited by the lack of a framework to examine the many variables that make up this continuum of influence.

## Methods

Based on reviews of scholarly literature on aid and health, power in health policy, and national health priority setting, we evolved a heuristic to understand the influence of private philanthropy in national health priority setting. The heuristic considers the properties of private philanthropy, categorizes the ways in which political resources are deployed (through direct *vs.* indirect, formal *vs.* informal mechanisms), and summarizes how influence may affect (a) the relative priority given to health *vs.* other domains, (b) across health domains, and (c) within a single domain of health. Drawing upon analysis of existing literature and secondary data from publicly available information, this heuristic was applied to develop case studies of two long-standing private philanthropies in India: the Sir Ratan Tata Trust (SRTT) and the Rockefeller Foundation (RF).

**Figure 1:**
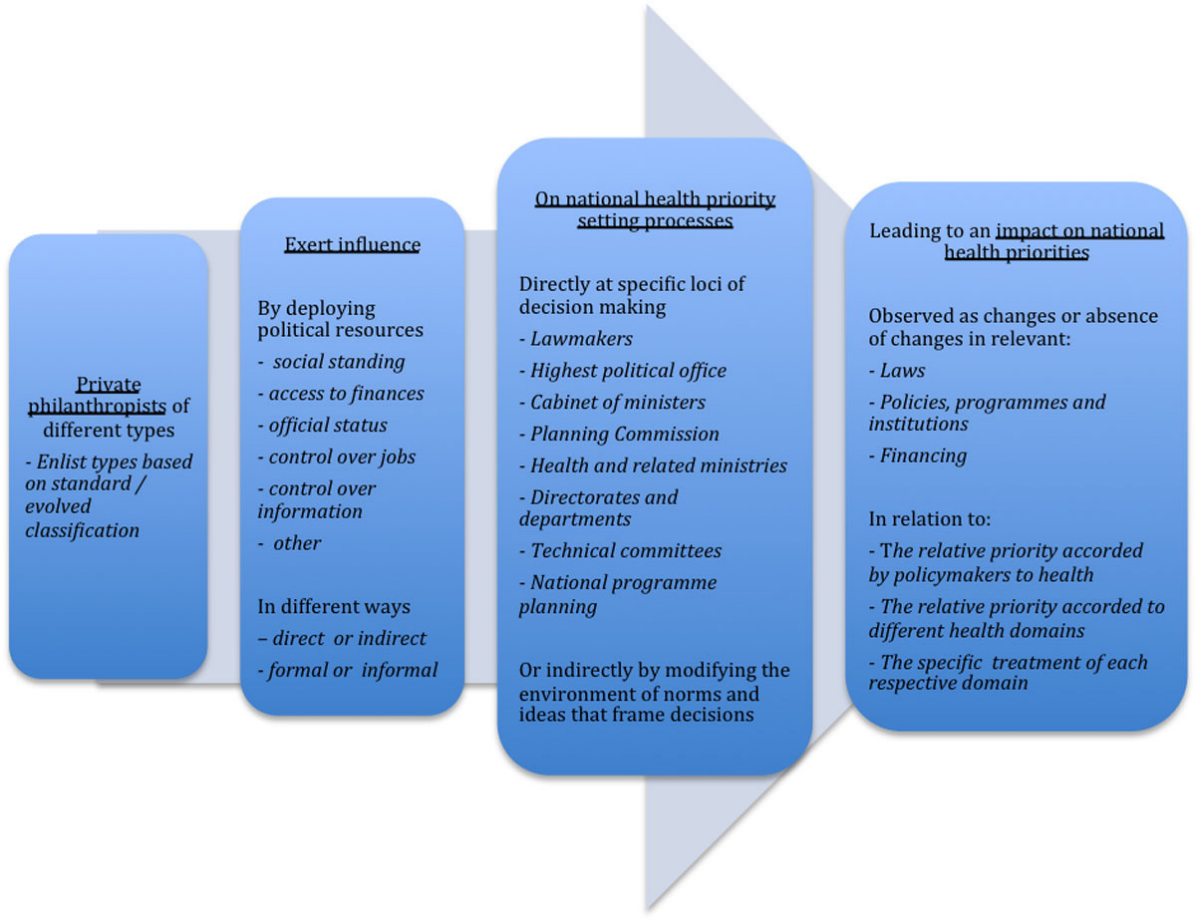
A heuristic to understand the influence of private philanthropy in national health priority setting

## Results

In applying our heuristic, we found that SRTT, a 93-year old family trust has, throughout its existence, encapsulated Jamsedji Tata’s vision of supporting “the most gifted, so as to make them of greatest service to the country.” Its chairman has legitimized the role of business leaders in the health domain, while the trust has prioritized rural and tribal health, mental health and disability, human resources and facilities upgradation, among other issues that have historically been under-prioritized in Indian health policymaking. As per Jamsedji’s motto, SRTT’s support is indirect: it funds recognised individual and organisations active in health policymaking with a community health orientation.

RF was established in 1913 “to promote the well being of humanity around the world”. RF support for hookworm control in post-independence India accorded priority to the health sector, putting this otherwise unknown disease onto the country’s health agenda. It also directly promoted a vertical, technological approach to disease control, including India in its international agenda to demonstrate the efficacy of its ‘cure’. This set a precedent for its funding-driven involvement in vertical health policy planning in India. More recently, moreover, RF has used its position of influence to directly support health systems strengthening in India.

## Discussion

The peculiar histories and financial arrangements of each foundation belied functional categorisation; this was a challenge in applying our heuristic. However, we could identify specific approaches adopted by foundations to influence Indian health priority setting.

We found that SRTT’s influence is intended to fill gaps left by the public system and as such, does not directly engage with it. The foundation does provide support to established civil society actors so that they may influence policymaking.

In contrast to SRTT, RF has directly funded single disease technological initiatives, making them priorities on India’s health agenda. More recently, RF has directly supported health systems agenda-setting activities and research related to universal health coverage.

Our study shows that giving can influence governance directly and indirectly in the priority accorded to health, the prioritization of topics within health, and models of funding in health. The context of SRTT may well be different from that of other private players in the large and growing ecosystem of giving in India.

Further application of this heuristic may enable comparison of the influence of private players we studied as well as the role other players (diaspora foundations, religious charities, as well as national and transnational corporate social responsibility platforms) have in priority setting.

## Competing interests

Authors declare that they have no conflict of interest.

## Funding statement

The study was funded by the Oxfam India.

